# Recent advances in the management of liposarcoma

**DOI:** 10.12688/f1000research.10050.1

**Published:** 2016-12-22

**Authors:** Nadar A. Nassif, William Tseng, Camille Borges, Peter Chen, Burton Eisenberg

**Affiliations:** 1 Hoag Family Cancer Institute, Hoag Hospital, Newport Beach, CA, USA; 2 Orthopedic Research and Education Institute, Sand Canyon Avenue, Irvine, CA, USA; 3 Hoag Orthopedic Institute, Sand Canyon Avenue, Irvine, CA, USA; 4 Department of Surgery, Section of Surgical Oncology, Keck School of Medicine, University of Southern California, San Pablo, Los Angeles, California, USA; 5 USC Norris Cancer Center, Los Angeles, California, USA

**Keywords:** liposarcoma, surgical resection, radiation therapy, palbociclib, soft tissue sarcoma

## Abstract

Liposarcoma is the most common soft tissue sarcoma. With its various subtypes, the natural history of this disease can vary significantly from a locally recurrent tumor to a highly malignant one carrying a poor prognosis. Progress in the understanding of the specific molecular abnormalities in liposarcoma provides greater opportunity for new treatment modalities. Although surgical resection and radiation therapy remain the keystones for the management of primary liposarcoma, the inclusion of novel agents that target known abnormalities in advanced liposarcoma enhances the potential for improved outcomes.

## Introduction

Liposarcoma represents the most common subtype of soft tissue sarcoma, comprising up to 20% of new diagnoses. The prevailing opinion is that the putative cell of origin is an adipocyte progenitor halted in its differentiation sequence. Within the classification of liposarcoma, there are subtle variants which lead to differences in disease pattern, treatment, and outcomes.

The goal of this article is to briefly review these subtypes of liposarcoma and discuss the latest in treatment options and the potential future direction for the management of this disease.

## Subtypes of liposarcoma

Liposarcoma has a spectrum of pathological variations that directly impact prognosis. The World Health Organization has classified liposarcoma into several subtypes ^1^. Chang *et al*., in a series of 127 patients, first demonstrated notable differences in disease-free and overall survival based on histologic subsets, and these clinical differences have remained consistent with our contemporary classification system ^2^.

Atypical lipomatous tumor (ALT) and well-differentiated liposarcoma (WDLS) represent a locally aggressive tumor with no risk of metastatic disease ^3^. This entity comprises the most common type of liposarcoma at around 40%. The term ALT is reserved for those tumors that arise in the extremities, whereas WDLS is more reserved for those tumors found in the retroperitoneum or mediastinum. However, both entities share identical histological features. Dedifferentiated liposarcoma can arise *de novo* but may represent a progression from pure WDLS to high-grade malignancy. This aggressive feature of dedifferentiation can occur at the time of local recurrence of WDLS ^3^.

Myxoid round cell liposarcoma (MRCL) is the second most common variant at around 20% of all lipogenic sarcomas. These tumors may have a hypercellular round cell component that portends a worse prognosis ^4^. It is suggested that round cell components above 25% indicate a high-grade neoplasm; however, there have been reports confirming a lower threshold of 5% as the cut off for high-grade tumors ^5^. Additionally, MRCLs have demonstrated a unique metastatic pattern with a propensity for fat-bearing areas (bone marrow, mediastinum, retroperitoneum, etc.) ^6– 8^. Schwab *et al*. reported that, of 230 patients with MRCL, 17% developed skeletal metastases with the most common sites being the spine and ribs. Non-skeletal sites included the lungs, abdomen, and retroperitoneum. It is, therefore, important to mention that restaging considerations for these patients include abdominal and pelvic imaging, as well as evaluation of the spine by magnetic resonance imaging for metastatic lesions, in addition to the more common routine local and pulmonary surveillance ^7, 9^.

Pleomorphic liposarcoma is the high-grade subtype. Fortunately, this subtype represents only 5% of all liposarcomas. Multiple studies have demonstrated rather poor outcomes with a 34–45% risk of local recurrence and 32–57% risk of metastatic disease ^10– 12^. A variant of WDLS termed “dedifferentiated liposarcoma” is more commonly present in the retroperitoneum. Similar to the pleomorphic type, these tumors present with a high local recurrence rate of 41% and high metastatic potential (17–30%) with a 5-year mortality rate of 28% ^13– 15^.

## Genetic biomarkers

Unique genomic abnormalities have been identified within liposarcoma subtypes and have been clinically useful for both diagnostic and therapeutic considerations. As diagnostic molecular pathology with comprehensive genomic profiling matures, specific markers will likely be incorporated into targets for opportunities in new drug development. Approximately 90% of WDLS/ALTs display a 12q12-15 amplicon creating a ring twelfth chromosome that represents amplified oncogenes *MDM2* and *CDK-4* ^16– 18^. Dedifferentiated liposarcoma is molecularly similar to WDLS/ALT with amplicons in 12q12-15 despite its inherent more-aggressive biological activity. The molecular mechanisms that contribute to the high-grade features of dedifferentiated liposarcoma have not been fully elucidated ^19, 20^.

MRCL is characterized by a recurring unique chromosome rearrangement, t(12;16)(q13;p11), resulting in a *TLS-CHOP* fusion oncoprotein that is present in 95% of cases. Rarely seen is another translocation fusion, *EWS-CHOP* oncogene *t(12;21) (q13;q12)*. These chromosomal abnormalities contribute to lipogenic arrest and are pathognomonic for MRCL ^21, 22^.

The pleomorphic variant demonstrates a diverse mix of chromosomal rearrangements and genomic profiles without unified alterations. The most common mutations seen are found in *p53* ^21^.

## Surgical management of liposarcoma

Surgical resection of liposarcoma in the extremity follows oncologic principles. A goal of a wide resection with a negative margin is always desired. ALTs are often intramuscular and do not typically invade bone. Primary resection of these tumors is usually uncomplicated; however, the goal of a complete resection is necessary to minimize the risk of local recurrence. Higher-grade subtypes such as MRCL and pleomorphic liposarcoma, depending on the extent and invasiveness of the mass, may require resections of entire muscle subgroups in order to allow for adequate margins. ****

Retroperitoneal liposarcomas are arguably much more challenging to treat from a surgical standpoint than are extremity liposarcomas ^23, 24^. Tumors in the retroperitoneum are frequently massive in size (median 30 cm) and can involve adjacent visceral organs and critical structures. Complete resection of the tumor is the standard of care, and obvious tumor invasion of adjacent organs or structures mandates resection. However, the optimal extent of resection is controversial, with some sarcoma centers advocating resection of adjacent organs or structures even without obvious evidence of tumor invasion ^25, 26^. This technique of extended or compartmental resection has been shown in retrospective studies to decrease locoregional recurrence rates and even improve overall survival for low- to intermediate-grade disease ^27^. Ultimately, the optimal extent of resection should also consider histologic subtype and balance the potential morbidity of surgery with expected oncologic outcome (e.g. high rate of distant metastasis for truly high-grade differentiated liposarcoma) ^28– 30^.

## Radiation therapy

Treatment of liposarcoma with radiation is informed by randomized trials in extremity sarcoma showing improved local control with adjuvant radiation following limb-sparing surgery ^31– 33^. For high-grade tumors of the extremity, decisions regarding dose, volume, and pre- or post-operative treatment can be approached similarly to other sarcoma histologies.

The role of radiation in the management of ALT is controversial. Cassier *et al*. of the French Sarcoma Group (Groupe Sarcome Français – Groupe d’Etude des Tumeurs Osseuses [GSF-GETO]) analyzed the Conticabase database and found that adjuvant radiation for extremity and trunk wall ALT/WDLS led to a 5-year local relapse-free survival of 98.3% versus 80.3% without radiation therapy (hazard ratio 0.26) ^34^. Although a local control benefit may be achieved with adjuvant radiation therapy, there is no expected survival benefit given that these tumors do not metastasize. Thus, care is individualized, considering the extent of surgery (patients with an R0 resection have a low risk of recurrence) as well as the risks of relapse to organ function and the morbidity and feasibility of further surgery.

Myxoid liposarcoma is highly radiosensitive, and dramatic responses with pre-operative radiation have been reported ^35, 36^. Pitson *et al*. of Princess Margaret showed a 59% tumor volume reduction after pre-operative radiation ^37^. This radiosensitivity has translated into excellent local control rates, with both Chung of Princess Margaret and Guadagnolo of MD Anderson reporting 97% local control with combined surgery and radiation ^38, 39^. The responsiveness of myxoid liposarcoma makes this tumor amenable to pre-operative radiation therapy, particularly in cases where upfront surgery may be difficult or morbid.

In the retroperitoneum, the role of adjuvant radiation therapy is evolving. Retrospective series as well as two prospective series have shown favorable overall survival and local control with adjuvant radiation therapy as compared to surgery alone ^40– 42^. In addition, radiation therapy may be delivered with acceptable toxicity, particularly with intensity-modulated radiation therapy and pre-operative therapy ^40, 43, 44^. Pre-operative radiation therapy is the preferred method of adjuvant radiation therapy for retroperitoneal sarcoma for the benefits of 1) displacement of bowel out of the radiation therapy field by the *in situ* tumor, 2) defining a more accurate volume, 3) theoretically reducing intra-operative tumor seeding, and 4) delivering an overall smaller radiation dose ^45^.

Based on the above results, the European Organisation for Research and Treatment of Cancer (EORTC) is currently conducting a randomized trial (EORTC 92092-22092; STRASS trial) comparing 50.4 Gy of pre-operative radiation therapy followed by surgery to surgery alone. With respect to retroperitoneal liposarcoma specifically, Ecker *et al*. performed a propensity score-matched cohort analysis of the US National Cancer Database and found that neoadjuvant radiation therapy led to an improvement in survival (median overall survival 129.2 versus 84.3 months, p=0.046, hazard ratio 1.54) ^46^. Thus, while results of the EORTC trial are awaited, patients with retroperitoneal liposarcoma may be considered for pre-operative radiation therapy.

## Systemic therapy

There is evidence of differential response and sensitivity to chemotherapy based on liposarcoma subtype ^47^. This differential response may also be important relative to anatomic site, with extremity liposarcoma (MRCL) responding better than other sites of origin. In metastatic disease, a traditional regimen containing doublets of doxorubicin/ifosfamide or gemcitabine/docetaxel result in response rates of 25 to 35% and survival of 12 to 18 months ^48, 49^. In terms of clinical application, MRCL appears to be the only subgroup of liposarcoma that is likely chemosensitive as measured by response rather than disease stability.

Several newer agents have become useful in consideration of patients with metastatic disease. Both trabectedin and eribulin have received recent FDA approval for application in the second-line setting for liposarcoma. Trabectedin seems to be particularly active in MRCL and may actually be considered for first-line therapy for selected MRCL patients ^50– 52^.

Targeted therapy for advanced liposarcoma has shown promise early on. Because of a general meaningful lack of chemotherapy response in WDLS and dedifferentiated liposarcoma, an attractive target is the *CDK4* oncogene, which is amplified in 90% of cases. Palbociclib, a potent *CDK4/CDK6* inhibitor, has shown activity in WDLS and dedifferentiated liposarcoma by halting disease progression ^53, 54^. Another potential avenue for targeted therapy in this liposarcoma subtype is the significant presence of the *MDM2* amplicon. RG7112 is an MDM2 antagonist that has shown activity in a small proof-of-principle study that warrants further evaluation ^55^.

Limited results investigating agonists of PPAR-gamma (regulator of adipocytic differentiation) have not proven particularly beneficial for advanced liposarcoma. Additionally, nelfinavir, a protease inhibitor used in HIV treatment and thought to contribute to treatment-related lipodystrophy through alteration of SREBP-1, a transcriptional regulator expressed in liposarcoma, has been the subject of a clinical trial. Thus far, this class of agents has shown no proven benefit ^56, 57^.

These early results of molecular target-specific therapy are intriguing but need further elucidation for efficacy and safety in larger patient trials. It may be that combination therapy or an optimized pharmacokinetic variant of a liposarcoma-specific oncoprotein-targeted drug will be necessary before survival is affected.

## Future directions

The increasing opportunities for new therapies are based on the activation/suppression of the tumor–host immune response. Tseng *et al*. described a unique adaptive immune response in WDLS or dedifferentiated liposarcoma which may have potential therapeutic implications ^58, 59^. In these tumors, organized aggregates of immune cells (known as tertiary lymphoid structures) have been observed, and, based on the cellular composition, these are likely sites of intratumoral antigen presentation. Tseng *et al*. also reported that the majority of tumor-infiltrating, effector CD8+ T cells have high expression of PD-1, which suggests that immune checkpoint inhibitors may have efficacy in this disease. Additionally, immune response stimulation utilizing the cancer-testis antigen NY-ESO-1 as a vaccine target may have a role in MRCL ^60^.

Investigation into the efficacy of the immune response in WDLS and dedifferentiated liposarcoma are ongoing and will be vital to develop new immunotherapeutic approaches to treatment ^61^.

## Conclusion

Liposarcoma encompasses a variety of soft tissue sarcomas across a biological continuum. This variety is characterized by differences in growth promoters and metastatic potential. The main treatment options for primary disease are surgical or a combination of surgery and radiation. Systemic treatment management has been improved somewhat by the approval of several new agents and the potential of targeted therapy through a more complete knowledge of the molecular genomic basis for this rare malignancy.
